# SENSE: A Sensor-Centric Multimodal Evidence Chain for Teaching Evaluation in Smart Classrooms

**DOI:** 10.3390/s26206617

**Published:** 2026-10-19

**Authors:** Lihong Cheng, Xiaohe Liu, Haisheng Yu

**Affiliations:** 1 Department of Foundational Courses, Dalian Jiaotong University, Dalian 116028, China; chenglh@djtu.edu.cn; 2 School of Art and Design, Dalian Jiaotong University, Dalian 116028, China; 3 China Internet Network Information Center, Beijing 100070, China

**Keywords:** smart-classroom sensors, multimodal sensor fusion, teaching evaluation, learning analytics, large language models, evidence-grounded evaluation

## Abstract

Smart-classroom teaching evaluation increasingly relies on heterogeneous sensing sources, including classroom cameras, microphones, speech-recognition systems, and learning-platform interaction logs. Existing systems often treat these streams as separate analytical inputs, with insufficient attention to cross-modal alignment, missing-sensor robustness, and evidence-grounded large-language-model reasoning. This paper proposes SENSE (Sensor-Evidence Network for Smart-Classroom Evaluation), a sensor-centric framework that organizes classroom evaluation as an end-to-end evidence chain. Visual behavior, acoustic-semantic content, and learning-platform events are aligned in lesson-level time windows and converted into structured evidence packets. Available learning-outcome anchors provide contextual evidence to the semantic evaluator, whereas expert ratings are retained exclusively as external validation references. A DeepSeek-based semantic evaluator then generates engagement, instructional-content, interaction-quality, and teaching-feedback judgments under a fixed output schema. The framework was evaluated using 48 college-English lessons, 48 videos, 48 audio recordings, 4628 timestamped learning-platform interaction logs, and 960 valid student-window observation instances. Compared with a transparent rule-based operational baseline, SENSE reduced the engagement-evaluation error from 12.3% to 5.8%, reduced the content-coverage error from 14.5% to 6.2%, and increased the correlation with the mean expert score from 0.71 to 0.89. Under 30% random evidence removal, the correlation remained 0.80. The results indicate that teaching evaluation benefits from a multimodal sensor-evidence and semantic-reasoning process rather than a single behavior-recognition task.

## 1. Introduction

Smart classrooms make teaching processes observable at a level that is difficult to achieve through occasional classroom observation or end-of-term questionnaires. Cameras capture posture, head orientation, and visible participation; microphones and speech-recognition systems capture lectures and classroom discourse; and learning platforms record questioning, answering, and task participation. These sources make it possible to evaluate classroom teaching as a continuous evidence process rather than as a single subjective judgment.

Having more sensor data, however, does not automatically produce more credible evaluation. Visual behavior is observable but pedagogically incomplete. A high head-up rate, for example, is not equivalent to cognitive engagement and becomes meaningful only when interpreted together with instructional content, classroom interaction, and learning outcomes. Acoustic transcripts provide semantic information but cannot fully describe silent attention or off-task behavior. Interaction logs provide direct event evidence but may be sparse and dependent on teaching style. Consequently, classroom evaluation requires coordinated evidence alignment rather than simple feature concatenation.

Large language models (LLMs) offer a practical bridge between low-level sensing and high-level teaching judgment because they can compare lecture content with objectives, interpret interaction patterns, and generate diagnostic feedback. Their use also requires a reproducible evaluation protocol: sensor inputs, prompt versions, model settings, output schemas, and validation references must be specified so that the resulting judgments can be interpreted and checked against educational evidence.

This paper proposes SENSE (Sensor-Evidence Network for Smart-Classroom Evaluation), a framework that integrates multimodal sensing, cross-modal evidence alignment, constrained LLM reasoning, mixed-evidence validation, and missing-sensor analysis. SENSE does not directly map raw video and audio to teaching scores. Raw streams are first transformed into compact evidence units, aligned by lesson-level time windows, interpreted through a fixed semantic protocol, and validated against experts and learning outcomes.

The contributions are threefold:

(1) A unified sensor-evidence chain is developed to connect visual, acoustic-semantic, and learning-platform interaction-log evidence with teaching-level judgment and feedback.
(2) A structured multimodal evaluation protocol is introduced in which observable behavior is interpreted together with instructional semantics, learning-platform interaction logs, and outcome anchors, with explicit evaluation under missing-sensor conditions.
(3) An empirical evaluation protocol combines expert ratings, learning-outcome anchors, modality ablation, and missing-sensor stress tests to assess educational agreement and robustness.

## 2. Related Work

### 2.1. Sensor-Based and Multimodal Classroom Analytics

Smart-classroom research has widely adopted cameras, pose estimation, speech sensing, wearables, and learning-platform logs to analyze participation and teaching processes. Recent sensor and multimodal-learning-analytics studies show that classroom systems are moving from single devices toward multi-sensor data fusion and deployable evidence pipelines [1,2,3,4,5,6]. At the same time, real-world deployment studies emphasize persistent barriers involving sensing cost, synchronization, missing observations, privacy protection, sustainability, and data-governance accountability [7,8,9,10,11]. Vision-based methods can identify head orientation, posture, hand raising, writing, instructional activities, and other visible classroom behaviors [12,13,14,15,16,17]. For example, Chen et al. proposed a unified body-orientation framework for authentic classroom engagement analysis, showing that orientation can be a robust non-intrusive cue in crowded classrooms [12]. Such work is close to the visual-evidence component of SENSE, but it does not by itself evaluate teaching-content coverage, platform-mediated interaction, or agreement with expert and outcome references.

Multimodal learning analytics extends this perspective by jointly examining behavior, speech, text, environmental signals, and activity traces [18,19,20]. Two recent studies are especially relevant. Hou et al. proposed a text-centered multimodal attention model for classroom discourse quality using transcript, audio, and video streams, and reported that text dominated discourse assessment while acoustic evidence improved agreement with human ratings [21]. Fütterer et al. validated automated teaching-effectiveness scores from video, audio, and transcripts against expert ratings and achievement evidence in the Global Teaching Insights dataset [22]. Together with broader reviews of teacher-evaluation prediction in higher education [23], these studies establish strong state-of-the-art analogs for multimodal teaching evaluation. SENSE differs by treating classroom evaluation as an evidence-chain process that also includes timestamped learning-platform logs, constrained LLM-based semantic judgment, and missing-sensor stress testing.

A central limitation across sensor analytics is the gap between observable features and educational meaning. Head pose may provide a reproducible participation proxy, but it does not directly measure cognitive attention. Silence may indicate concentration during reading, disengagement during discussion, or compliance during explanation. Interaction events can reflect productive participation, but high event counts can also represent fragmented or low-quality exchanges. SENSE addresses this gap by aligning different evidence types around the same instructional episodes and by treating learning outcomes and expert ratings as validation anchors rather than as sensor modalities.

Teacher-facing dashboard and feedback studies further indicate that analytics become educationally useful only when they are interpretable, timely, and connected to teachers’ feedback practices [24,25,26,27,28]. This also matches formative-feedback research, which stresses that feedback should be specific, actionable, and focused on the learning task rather than on global judgment alone [29].

### 2.2. LLM-Assisted Teaching Evaluation

LLMs have been applied to educational question answering, grading, feedback generation, learner profiling, and classroom simulation, building on broader AI-in-education work on prediction, assessment, tutoring, and personalization [30,31,32,33,34,35]. Recent grading studies show that model choice, prompts, rubrics, and examples substantially affect scoring consistency [36,37,38]. These findings are relevant to teaching evaluation because classroom transcripts and structured interaction summaries require semantic comparison rather than only surface keyword matching. Table 1 summarizes the positioning of SENSE relative to closely related studies.

**Figure 1:**
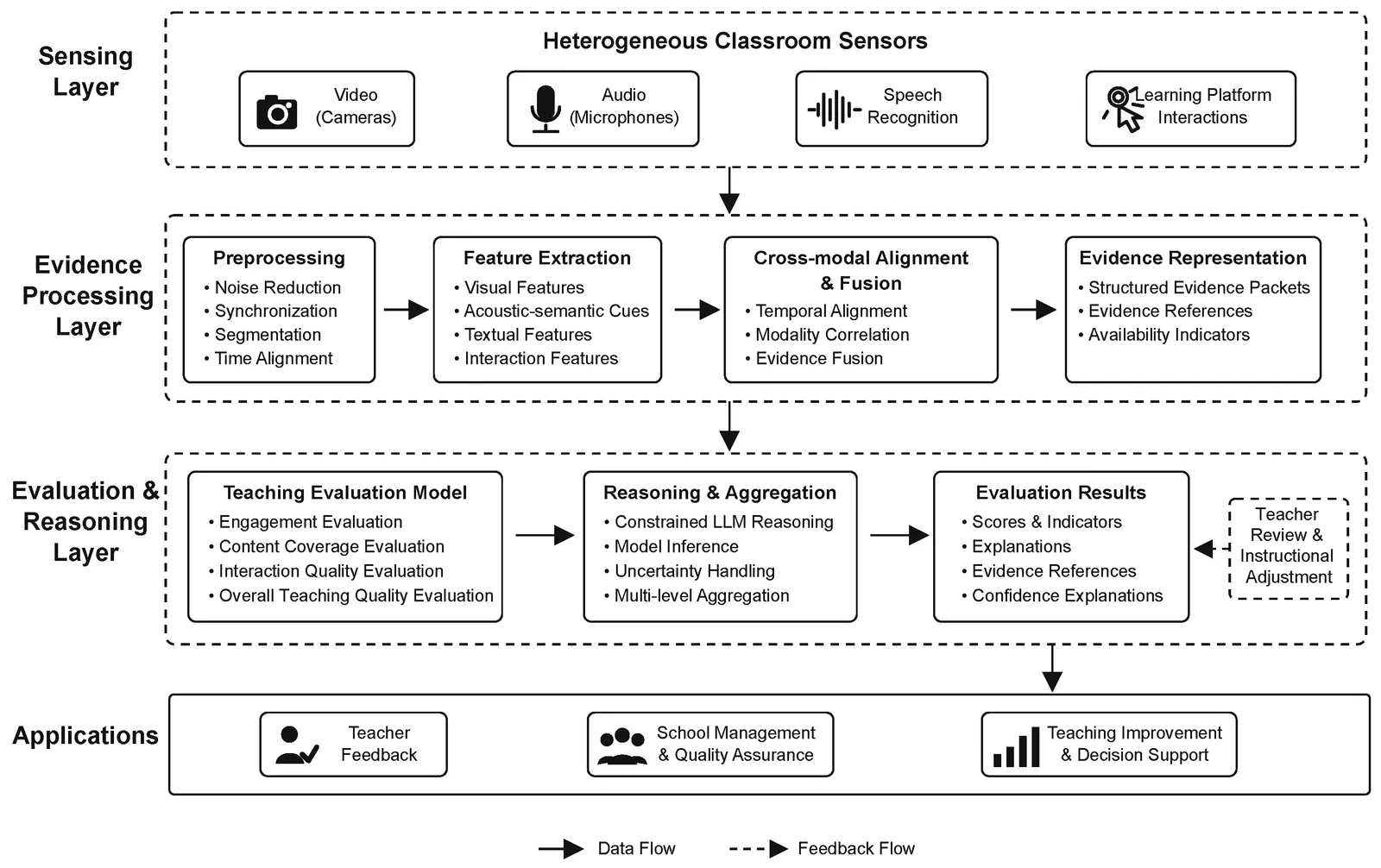
Sensor-to-evidence architecture of SENSE. Video, audio, speech-recognition transcripts, and learning-platform interaction logs are transformed into aligned evidence packets for constrained semantic evaluation, aggregation, and teacher-facing feedback. Outcome anchors enter as external educational references rather than as sensor modalities.

The same literature also cautions against treating free-form LLM outputs as directly reproducible measurements. Replicable educational use of LLMs requires transparent model identifiers, prompt versions, temperature settings, evidence inputs, rubric definitions, and human oversight [31,34]. SENSE therefore constrains the LLM to a fixed evidence schema, requires explicit references to evidence blocks, and records prompt and model identifiers. Figure 1 presents the overall sensor-to-evidence architecture of SENSE.

## 3. SENSE Methodology

### 3.1. Architecture and Evidence Definition

SENSE contains a sensing layer, an evidence-processing layer, and an evaluation-and-reasoning layer. The main workflow is

rawstreams→sensorevidence→alignedtuples→semanticjudgment→teachingfeedback.

For lesson window *t*, the aligned evidence tuple is

(1) $$X_{t}=\left\{V_{t},A_{t},I_{t},O_{t}\right\},$$

where $V_{t}$ denotes visual behavioral evidence, $A_{t}$ denotes acoustic-semantic transcript evidence, $I_{t}$ denotes learning-platform interaction-log evidence, and $O_{t}$ denotes available outcome-anchor evidence. Expert ratings are not included as model inputs; they are retained as external references for validation.

### 3.2. Sensor-to-Evidence Transformation

Visual streams are transformed into observable behavioral indicators, including head-up/head-down patterns, active and passive participation, off-task behavior, disruptive behavior, temporal state changes, and observable-frame coverage. These indicators are treated as reproducible participation evidence rather than as direct measurements of cognition.

Audio is processed through automatic speech recognition and segmented into lecture, questioning, response, and activity intervals. The resulting acoustic-semantic evidence contains key concepts, teaching objectives, discourse focus, classroom questions, and semantic relations between taught and assessed content. In this manuscript, learning-platform interaction logs refer to timestamped events generated by the institutional smart-classroom or learning-management platform, including teacher-question release, student-response submission, answer selection, task submission, quiz completion, teacher-feedback release, and platform-recorded activity participation. These logs are distinct from observer notes and from spoken interaction inferred from the audio transcript. They are converted into event counts, response density, timing, activity submissions, and distributions across lesson stages.

Learning outcomes and expert evaluations are handled separately. Outcomes provide related evidence about whether the inferred teaching-content alignment is educationally meaningful. Expert ratings provide the reference criterion for evaluating the automated teaching judgments and are not used as LLM inputs.

### 3.3. Visual-Sensor Processing and Annotation

The visual-processing module produces window-level student behavior states and coverage indicators from sampled classroom frames. SENSE requires timestamped visual observations and a common aggregation window, but it does not require a specific camera model, detector architecture, frame rate, or window length. These implementation parameters are reported in Section 4 for reproducibility.

Student regions are detected and tracked across sampled frames by the classroom-analytics system. Head orientation is estimated from visual appearance features through a perspective-*n*-point head-pose calculation. A frame is treated as head-up when the absolute yaw angle does not exceed the configured yaw threshold and the pitch angle remains within the configured pitch range. Frames outside these ranges are classified as head-down or off-screen only when the face and upper-body region remain sufficiently visible.

Behavioral states are assigned at the analysis-window level:

- Active engagement: visible orientation toward the instructional activity accompanied by a speaking turn, hand-raising event, platform interaction, or teacher–student response;
- Passive engagement: visible orientation toward instruction without an observable interaction event;
- Disengagement: off-task, head-down, or off-screen behavior occupying more than the configured proportion of valid frames in the window;
- Disruptive behavior: behavior that interrupted or distracted the classroom process.

Occluded or unobservable students are not automatically classified as disengaged. A student-window observation is marked as missing when valid-frame coverage falls below the configured coverage threshold. Observable-frame coverage is reported separately from behavioral-state proportions.

For student *i* in window *t*, the state is encoded as a one-hot vector $e_{i,t}$. The class-level distribution is

(2) $$E_{t}=\frac{1}{N_{t}}\sum_{i=1}^{N_{t}}e_{i,t},$$

and the lesson-level distribution is

(3) $$E_{\mathrm{lesson}}=\frac{1}{T}\sum_{t=1}^{T}E_{t},$$

where $N_{t}$ is the number of observable students and *T* is the number of valid windows.

### 3.4. Cross-Modal Alignment and Semantic Evaluation

A windowing-and-alignment module divides each lesson into non-overlapping temporal windows after receiving the lesson start time, lesson end time, and configured window length. For window $t=[s_{t},e_{t})$, visual states are assigned by frame timestamp, transcript segments are assigned by maximum temporal overlap or split at the boundary when needed, and learning-platform log events are assigned by event timestamp. Outcome anchors are attached at the lesson or sequence level rather than to individual frames. Each evidence tuple retains modality-availability flags and missingness indicators, so the semantic evaluator can distinguish “not observed” from “observed as absent”.

This alignment prevents multimodal evaluation from becoming a simple concatenation of unrelated features. For example, a vocabulary-explanation window may combine rising passive-engagement levels, transcript evidence covering target concepts, and low response density in the same instructional episode. The resulting tuple supports a specific teaching judgment rather than a generic comment.

Content coverage is estimated from semantic consistency between the lesson transcript *L* and assessment or activity content *Q*:

(4) $$C={\mathrm{sim}}_{\mathrm{sem}}\left(\phi (L),\phi (Q)\right),$$

where ϕ(·) maps text to structured knowledge-point representations and ${\mathrm{sim}}_{\mathrm{sem}}$ measures conceptual rather than lexical overlap.

Semantic evaluation was performed through the DeepSeek API using the deepseek-chat model identifier recorded at execution time. The model was used in non-thinking mode with a temperature of 0.1, top_p = 1.0, a maximum output length of 2048 tokens, JSON-constrained output, and streaming disabled. Each lesson was evaluated three times using the same evidence packet. Continuous scores were aggregated using the median and categorical judgments using majority agreement.

The prompt contained four blocks: visual evidence, acoustic-semantic evidence, learning-platform interaction-log evidence, and outcome anchors. The required output schema contained engagement, content coverage, interaction quality, overall teaching quality, evidence references, feedback, and a confidence explanation. Every judgment was required to cite at least one evidence block. The prompt template, scoring rubric, JSON schema, and prompt-version identifier are provided in Supplementary Material S1.

Only de-identified lesson-level summaries were transmitted to the API for semantic evaluation. Raw video, raw audio, facial images, names, student identifiers, teacher identifiers, and individual-level records were not transmitted externally. The AI service was used only as a semantic evaluation component and did not process raw human-participant data.

### 3.5. Mixed-Evidence Validation

**Figure 2:**
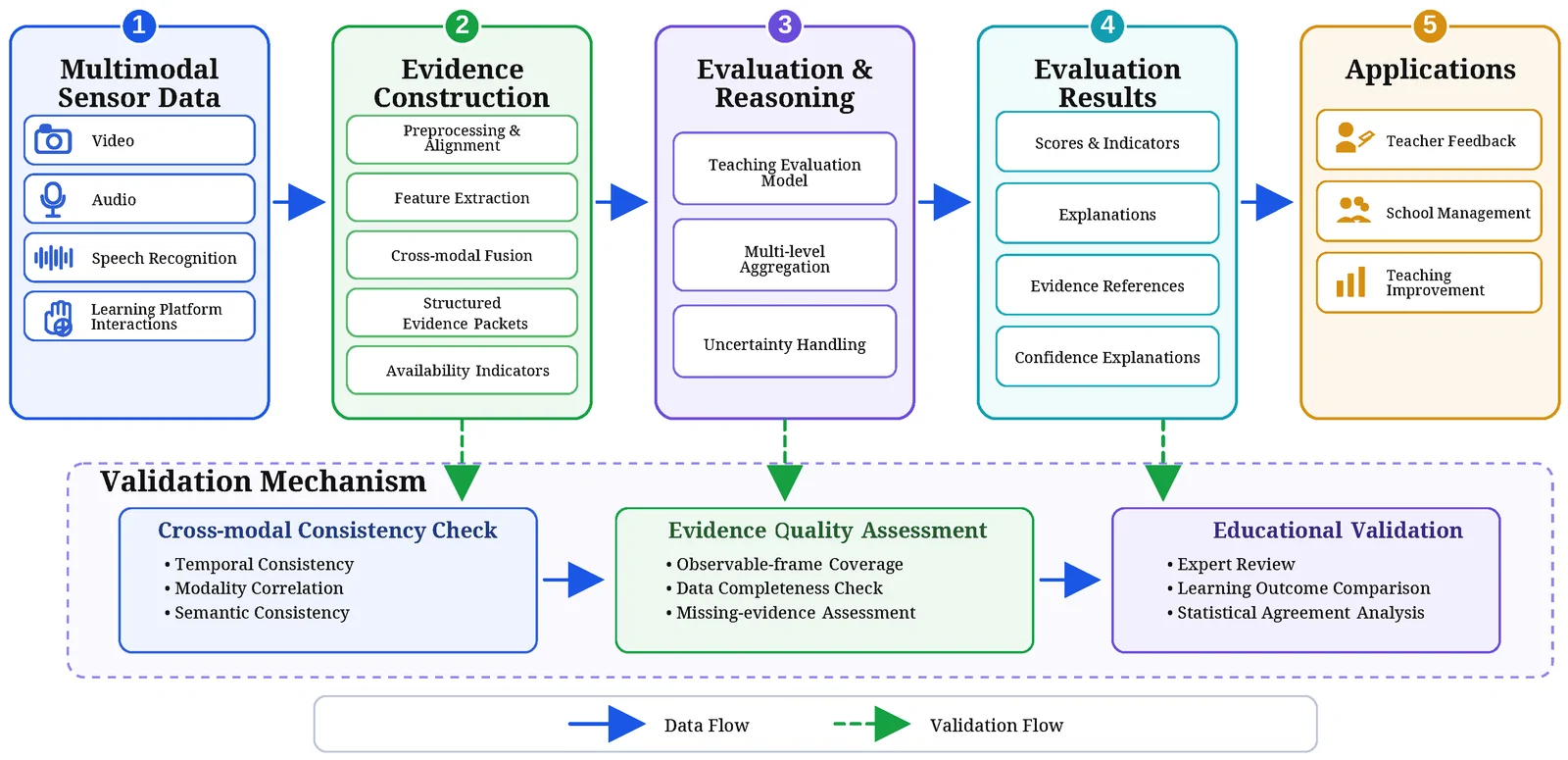
Mixed-evidence validation framework of SENSE, comprising checks on aligned multimodal evidence, evidence quality and availability, and educational comparison with expert ratings and learning outcomes.

SENSE uses expert teaching-evaluation scores, sensor-derived classroom indicators, and learning outcomes as complementary validation sources. As illustrated in Figure 2, the validation framework distinguishes checks on aligned multimodal evidence, evidence quality and availability, and educational comparison with expert ratings and learning outcomes. The modality-specific and fused configurations are compared in the ablation analysis, while missing-evidence experiments assess the effect of incomplete inputs. This separates the observable sensor evidence used by the system from the external educational references used to assess whether the resulting judgments are meaningful.

## 4. Experimental Design

### 4.1. Dataset and Evaluation Protocol

The data were collected from routine college-English teaching, classroom observation, teaching questionnaires, and teaching-evaluation activities at Dalian Jiaotong University. The dataset contains 48 lessons, each lasting approximately 45 min, including 48 classroom videos, 48 audio recordings, 4628 timestamped learning-platform interaction logs, and 960 valid student-window observation instances. The primary unit of analysis was the lesson, resulting in 48 integrated evaluation samples.

A student-window observation instance refers to one de-identified student’s visual-behavior state in one 30-s lesson window after quality filtering. A student-window was retained when at least 50% of the expected sampled frames were valid and could be aligned with the lesson timeline. These 960 instances were used only to construct aggregated lesson indicators, such as engagement-state proportions and observable-frame coverage. They were not treated as statistically independent samples, and no individual-level observation was transmitted to the LLM service.

For this experiment, classroom videos were sampled at 2 frames per second and divided into 30-s non-overlapping analysis windows. The yaw and pitch thresholds for head-up classification were $35^{\circ }$ and $-20^{\circ }$ to $25^{\circ }$, respectively; disengagement was assigned when off-task, head-down, or off-screen behavior occupied more than 60% of valid frames in a window. These parameter values describe the present implementation rather than necessary constants of the SENSE framework. Two trained annotators independently labeled 480 randomly selected windows from 12 lessons to assess the visual-state definitions. Inter-annotator reliability was assessed with Cohen’s κ, and visual-state classification was checked using precision, recall, macro-F1, and a four-class confusion matrix. No new visual neural network architecture was introduced in this study; the institutional classroom-analytics system provided the detector-tracker outputs used by SENSE.

The de-identified analysis dataset was organized at the lesson level. Stable instructor, course-section, and class-group identifiers were not retained for modeling, so clustering by teacher or student group and held-out-teacher validation could not be estimated in the present study. We therefore interpret the results as internal lesson-level validation and report this limitation explicitly in the Discussion.

Five university English teachers, with an average of 11 years of teaching experience, evaluated the same 48 lessons rather than being assigned disjoint lesson subsets. The experts were not shown SENSE outputs or baseline outputs. Their rubric contained four analytic dimensions and one overall judgment, as summarized in Table 2. Each dimension was scored on a five-level ordinal scale and linearly normalized to 0–1 before averaging. For each lesson, the expert-reference score was the mean of the five normalized expert ratings.

The prompt template, thresholds, evidence transformations, and scoring rubric were fixed before final lesson-level scoring and were not optimized against the final expert-reference scores. Because no separate external development and test set was available, the reported agreement with experts should be interpreted as internal validation rather than as evidence of generalization across institutions, teachers, courses, or student groups.

The study addressed four research questions:

**RQ1**  Does aligned multimodal evidence improve engagement evaluation over a rule-based head-up method?
**RQ2**  Does acoustic-semantic analysis improve teaching-content coverage evaluation over keyword overlap?
**RQ3**  Do expert and outcome anchors support the educational interpretation of automated evaluation?
**RQ4**  Which evidence sources contribute most to accuracy, and how robust is the evaluation when sensor evidence is missing?

### 4.2. Baselines and Metrics

The baseline design follows the comparison logic in recent classroom analytics rather than treating a single rule system as a state-of-the-art model. Because recent published systems differ in subject domain, language, data access, output labels, and raw-signal availability, they cannot be reproduced directly on the present de-identified college-English dataset. We therefore implemented transparent analog baselines aligned with the closest recent literature: a visual orientation baseline related to classroom engagement studies [12,13]; an acoustic-semantic baseline related to transcript-centered discourse assessment [21]; an interaction-log baseline related to smart-classroom teacher–student interaction indices [39]; and a visual-plus-acoustic multimodal baseline related to holistic automated teaching-effectiveness assessment [22]. The rule-based baseline used threshold-based head-up duration and interaction counts for engagement evaluation and transcript-assessment keyword overlap for content coverage; it is retained as an interpretable operational baseline, not as a claim about the best possible published method. The comparison therefore supports improvement over transparent and literature-aligned baselines under a common dataset constraint, but it is not presented as universal superiority over every recent published model.

For each lesson, the reported correlation was Pearson’s *r* between automated scores and the mean expert score. Spearman’s ρ was additionally examined when normality or linearity assumptions were not satisfied. The engagement and content errors were lesson-level mean absolute errors:

(5) $$\mathrm{MAE}=\frac{1}{n}\sum_{i=1}^{n}\left|{\hat{y}}_{i}-y_{i}\right|.$$

Percentage MAE values are reported in percentage points. Fisher-transformed 95% confidence intervals were calculated for the reported Pearson correlations. Inter-rater reliability among the five experts was assessed using a two-way random-effects, absolute-agreement, average-measures intraclass correlation coefficient, ICC(2,*k*), with a 95% confidence interval. For paired baseline–SENSE comparisons, the normality of lesson-level error differences was tested using Shapiro–Wilk. A paired-samples *t*-test with Cohen’s $d_{z}$ was used when normality was satisfied; otherwise, a Wilcoxon signed-rank test with rank-biserial correlation was used. The tests were two-sided with p<0.05, and nonparametric confidence intervals for lesson-level estimates were estimated from 10,000 bootstrap resamples of the 48 lesson-level samples.

### 4.3. Missing-Sensor and Ablation Experiments

Missing-sensor robustness was evaluated by randomly masking 10%, 20%, and 30% of evidence units and by fully masking visual or acoustic evidence. The system recomputed the evaluation from the remaining evidence and generated an updated confidence explanation. The predictive ablation sequence was *V*, V+A, V+A+I, and V+A+I+O.

### 4.4. Feedback and Outcome-Response Analysis

After each lesson, aligned visual, acoustic-semantic, and learning-platform interaction-log evidence was converted into a teacher-facing diagnostic summary. The summary highlighted low-engagement windows, weakly covered concepts, low-response intervals, and confidence limitations. Teachers reviewed the summaries and selected actions, such as additional questioning, re-explanation of weak concepts, and adjustment of activity timing. Subsequent lessons were processed using the same pipeline. These comparisons were treated as outcome-response anchors rather than standalone causal evidence.

For the outcome-response analysis, panel (a) reports normalized outcome scores, defined as the class mean score divided by the maximum possible assessment score and expressed on a 0–100 scale. Panels (b) and (c) summarize five consecutive observation points, denoted T1–T5. Each observation point corresponds to a lesson-level feedback cycle: “before” denotes the lesson evidence before teachers reviewed the SENSE-supported diagnostic summary, and “after” denotes the subsequent lesson after teachers selected and implemented adjustment actions. Interaction frequency is the count of valid learning-platform interaction-log events per lesson. Higher interaction frequency is not treated as automatically better; it is interpreted only together with relevance, timing, response distribution, and content alignment.

## 5. Results

### 5.1. Engagement and Sensor Coverage

**Figure 3:**
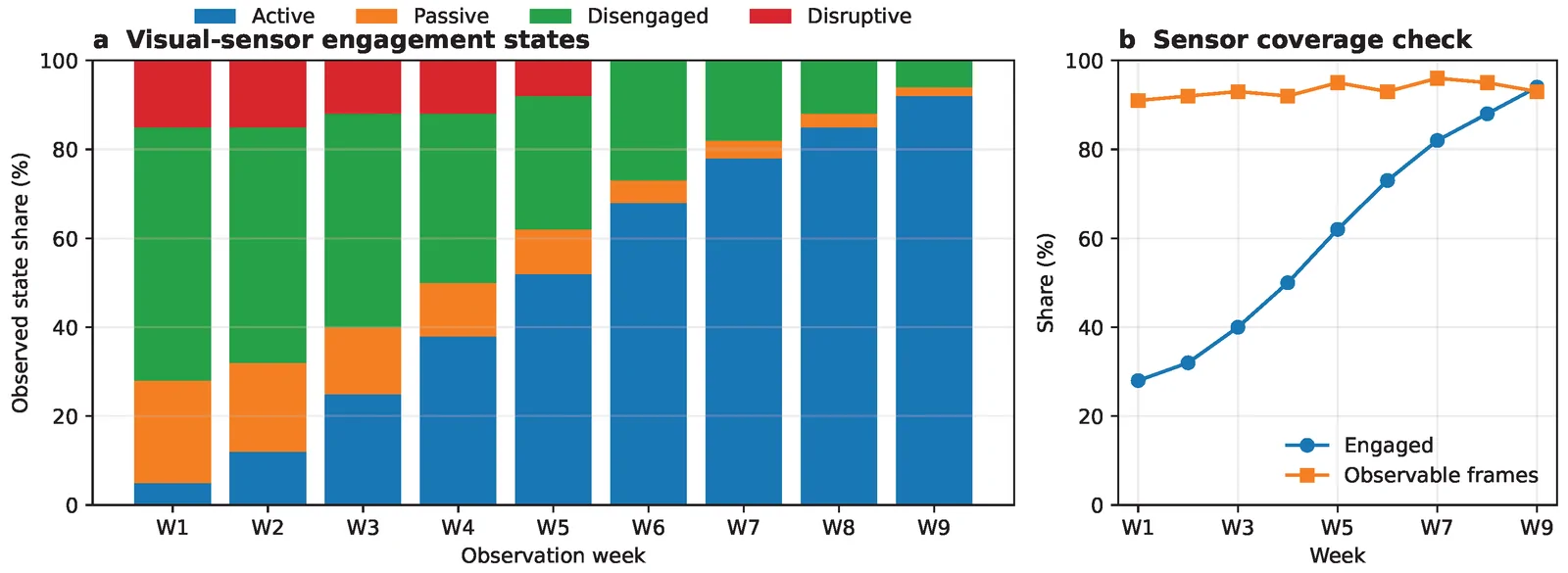
Visual-sensor engagement evidence over time. State distributions are shown together with observable-frame coverage.

Figure 3 shows the weekly distribution of active, passive, disengaged, and disruptive states together with observable-frame coverage. Active engagement increased across the observation period, while disengaged and disruptive states declined. Observable-frame coverage remained above 90%, indicating that the behavioral trend was not produced by a progressive reduction in valid observations.

The transparent rule-based operational baseline produced an engagement MAE of 12.3%, whereas SENSE reduced the error to 5.8%. The result supports RQ1: head orientation is useful as an observable cue, but its interpretation becomes more consistent with expert judgment when acoustic context and learning-platform interaction-log evidence are included.

### 5.2. Sensor-Modality Contribution

Table 3 reports the empirical modality comparison calculated from the 48 lesson-level samples. The visual-only model improved engagement estimation but retained high content error. The acoustic-semantic model reduced content error but did not fully capture classroom participation. The interaction-only evaluation was sensitive to sparse events. Visual-plus-acoustic fusion increased correlation with the expert mean to 0.84, while the complete evidence model reached 0.89 and produced the lowest error profile.

The mean absolute deviation from expert evaluation decreased from 0.118 for the rule-based baseline to 0.056 for SENSE on the normalized 0–1 scale. The paired lesson-level comparison was significant (p<0.01). The five experts achieved an average-measures ICC of 0.86, indicating strong human inter-rater reliability; the ICC confidence interval and the bootstrap intervals for prediction errors and paired differences are reported as part of the de-identified statistical summary supplied with the revision materials. Pearson’s *r* increased from 0.71 for the rule-based baseline (Fisher 95% CI: 0.53–0.83) to 0.89 for SENSE (Fisher 95% CI: 0.81–0.94). This model-reference correlation should not be interpreted as the same statistic as the human ICC, and the reported estimates should be read as internal lesson-level validation rather than external generalization evidence.

### 5.3. Outcome Anchors and Feedback Response

**Figure 4:**
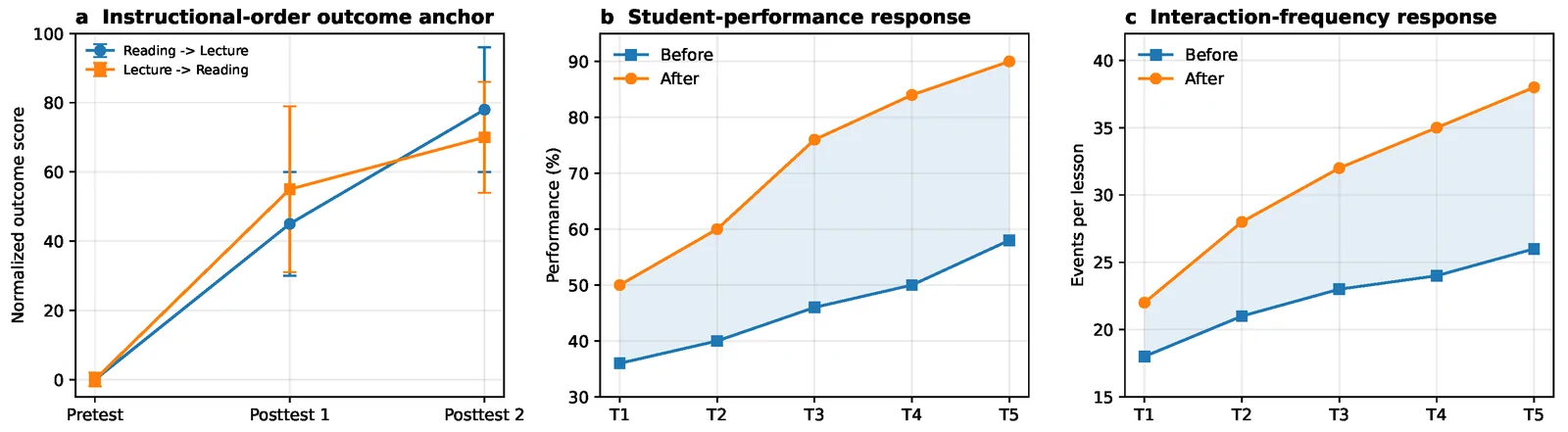
Learning-outcome and interaction responses associated with SENSE-supported feedback. (**a**) Normalized outcome-anchor comparison for two instructional orders; (**b**) student-performance response across five feedback cycles (T1–T5); and (**c**) learning-platform interaction-log frequency across the same cycles. In panels (**b**,**c**), before denotes the lesson before teachers reviewed SENSE feedback, and after denotes the subsequent lesson after teacher adjustment. The results are interpreted as outcome-response anchors rather than standalone causal evidence.

Figure 4 combines the instructional-order outcome anchor with before–after teaching responses. Panel (a) reports normalized outcome scores for two instructional orders: reading-to-lecture and lecture-to-reading. The plotted percentages represent class mean assessment scores divided by the maximum possible score. The two instructional orders produced different post-test trajectories, supporting the use of outcomes as contextual anchors for transcript-derived content coverage. Panels (b) and (c) show five consecutive lesson-level feedback cycles (T1–T5). For each cycle, “before” refers to the lesson evidence before teachers reviewed the SENSE-supported diagnostic summary, and “after” refers to the subsequent lesson after teachers implemented selected adjustments. Across the five observation points, mean student performance increased from 46.0% to 72.0%, an absolute change of 26.0 percentage points, while valid learning-platform interaction-log frequency increased from 22.4 to 31.0 events per lesson. The interaction-frequency result is interpreted together with relevance and timing; more events are not assumed to be pedagogically better by themselves. These descriptive changes are associated with SENSE-supported feedback but do not establish an isolated causal effect.

### 5.4. Ablation and Missing-Sensor Robustness

**Figure 5:**
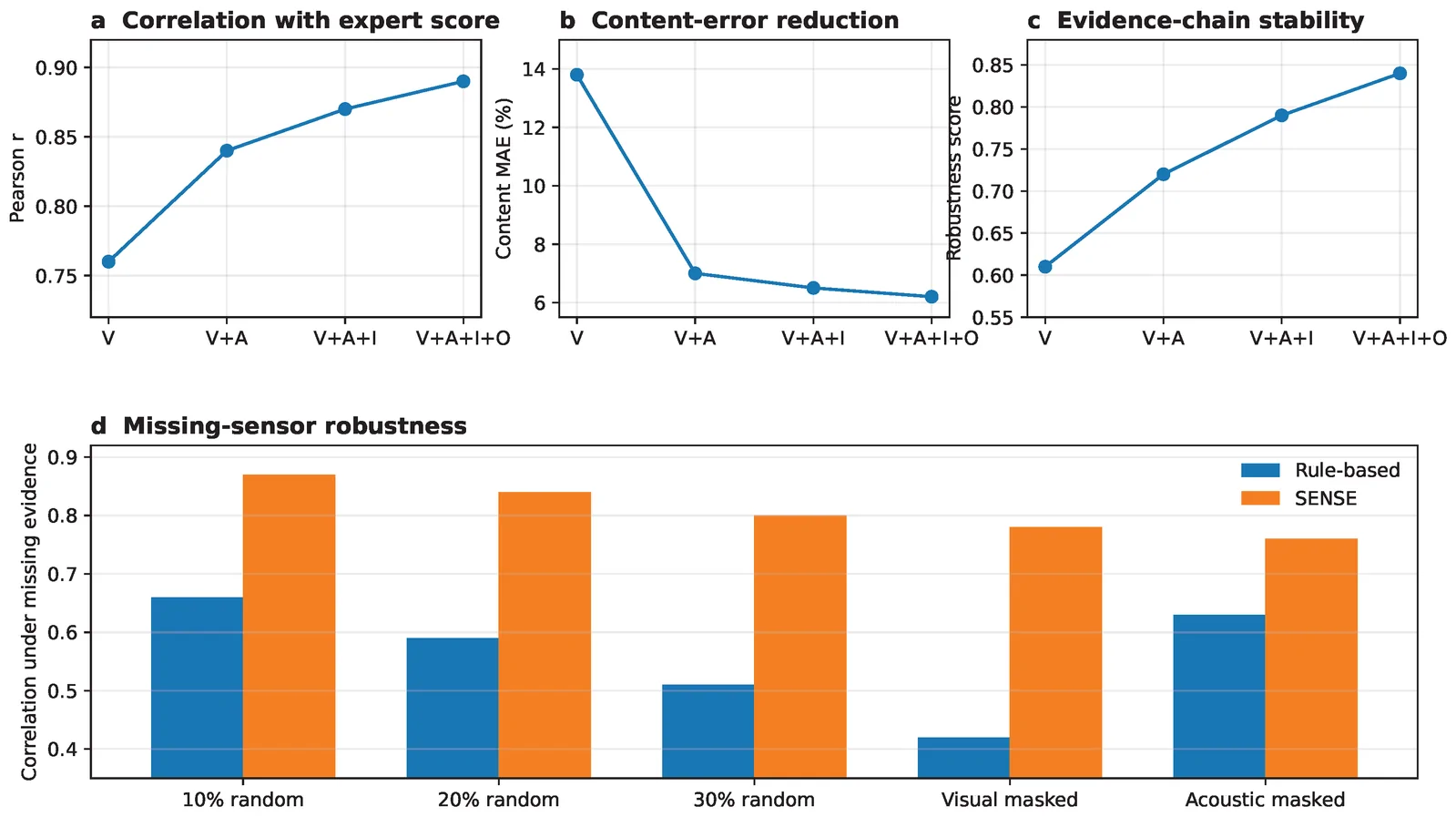
Evaluation-model ablation and robustness under missing sensor evidence. The predictive chain contains visual evidence (V), acoustic-semantic evidence (A), learning-platform interaction logs (I), and outcome anchors (O).

The ablation results in Figure 5 clarify the role of each evidence source. Adding acoustic semantics produced the largest reduction in content error. Learning-platform interaction logs improved the distinction between visible orientation and active participation, while outcome anchors connected process observations with educational results.

Table 4 shows that SENSE degraded more gradually than the rule-based baseline. With 30% random evidence removal, SENSE retained a correlation of 0.80, compared with 0.51 for the baseline. The largest SENSE degradation occurred when acoustic evidence was masked, confirming the importance of semantic information for content-related judgment.

## 6. Discussion

The revised study positions SENSE as an evidence-chain architecture rather than as a new detector, fusion network, or LLM. This positioning is important because recent work has already made strong progress in visual engagement analysis, multimodal discourse assessment, and automated teaching-effectiveness scoring [12,21,22]. The contribution of SENSE is to connect these otherwise separated functions into a reproducible workflow in which sensor observations, semantic interpretation, external validation, and missing-sensor robustness are reported as distinct but linked components.

For RQ1, the results indicate that aligned multimodal evidence improved engagement evaluation compared with the transparent rule-based head-up baseline. This finding is consistent with recent classroom-engagement studies showing that visual orientation is informative in authentic classrooms [12,13]. However, the reduction in engagement error from 12.3% to 5.8% suggests that orientation should not be interpreted alone. Acoustic context and learning-platform interaction logs helped distinguish passive attention, active participation, and low-response episodes.

For RQ2, acoustic-semantic evidence made the largest contribution to teaching-content coverage. The acoustic-semantic-only model reduced content error relative to visual-only and interaction-only settings, and the complete SENSE model reduced content error to 6.2%. This result agrees with Hou et al., who found that text is central in classroom discourse assessment and that additional modalities improve consistency with human judgment [21]. In SENSE, transcript evidence is not used as a standalone score; it is linked to objectives, activities, assessment content, and window-level classroom behavior.

For RQ3, expert ratings and outcome anchors supported the educational interpretation of automated evaluation, but the evidence should be read cautiously. The expert mean provided a human reference criterion, while outcome anchors showed whether content-coverage judgments were aligned with subsequent assessment patterns. This approach is compatible with recent multimodal teaching-effectiveness validation, where automated scores are compared not only with human ratings but also with student-learning evidence [22]. At the same time, the outcome-response analysis in Figure 4 is descriptive and cannot isolate the causal effect of SENSE feedback from teacher implementation, time, or student differences.

For RQ4, the ablation and missing-sensor experiments show that evidence sources contributed different strengths. Visual evidence was most directly related to engagement, acoustic-semantic evidence was most important for content coverage, interaction logs helped identify active participation, and outcome anchors connected process evidence with educational consequences. The gradual degradation under random evidence removal indicates that the aligned evidence design can continue to produce useful estimates when part of the sensor evidence is unavailable, although complete removal of acoustic evidence produced the largest loss for content-related judgment.

The study has several limitations. First, the data were collected from college-English lessons at one university and contain 48 lesson-level samples. External validation across institutions, disciplines, classroom layouts, and student populations is required. Second, the de-identified analysis files available for this revision did not retain stable instructor or class-group identifiers, so clustering and held-out-teacher validation could not be estimated. Third, head orientation remains a behavioral proxy rather than a direct measurement of cognitive engagement. Fourth, the performance of the semantic evaluator depends on ASR quality, prompt design, model version, and evidence completeness. Future work will expand the dataset, retain privacy-preserving grouping identifiers for hierarchical validation, compare multiple LLMs and supervised multimodal baselines, publish a fully de-identified replication package, and conduct controlled longitudinal studies.

## 7. Conclusions

This paper presented SENSE, a sensor-centric multimodal evidence chain for teaching evaluation. The framework aligns visual behavior, acoustic-semantic content, learning-platform interaction logs, expert references, and outcome anchors; constrains LLM reasoning through structured prompts and outputs; and evaluates robustness under missing evidence. On 48 lesson-level samples, SENSE improved correlation with expert scores and reduced engagement and content errors relative to transparent operational and single-modality analog baselines. These findings provide a practical basis for classroom evaluation systems that are evidence-grounded, robust to incomplete sensor evidence, and capable of producing actionable teaching feedback. 

## Figures and Tables

**Table 1:** Positioning of SENSE relative to closely related recent studies and system components.

Study or Approach	Main Focus	Main Evidence or Method	Relationship to SENSE
Chen et al. [12]	Classroom engagement from body orientation	One-stage body detection and orientation estimation in authentic classrooms	Provides a strong visual-engagement analog; SENSE uses visual states as one evidence source rather than as the complete evaluation.
Hou et al. [21]	Classroom discourse quality	Text-centered multimodal attention and multi-task learning on transcript, audio, and video	Closest semantic multimodal analog; SENSE emphasizes evidence packets, LLM-constrained reasoning, and platform logs.
Fütterer et al. [22]	Automated teaching- effectiveness assessment	Multimodal ML scores validated against experts and student achievement	Closest holistic teaching-evaluation analog; SENSE adds an explicit sensor-evidence chain and missing-sensor evaluation.
Ma et al. [39]	Teacher–student interaction index	Delphi and AHP-based smart-classroom interaction indicators	Supplies an interaction-rubric analog; SENSE operationalizes timestamped platform logs and aligns them with video and transcript evidence.
Tuo et al. [40]	Smart-classroom personalized-learning measurement	Validated questionnaire/instrument for pedagogy, space, and technology	Informs the interpretation of learning outcomes and feedback response but is not an automated multimodal system.
SENSE	Evidence-grounded teaching evaluation	Visual, acoustic-semantic, learning-platform, outcome, and expert evidence	Integrates established components into a reproducible evidence-chain architecture; it does not claim to introduce a new detector or LLM.

**Table 2:** Expert teaching-evaluation rubric used as the external reference. The full rubric and examples are provided in Supplementary Material S1.

Dimension	Evaluation Focus	Normalization
Engagement evidence	Visible attention, active participation, off-task behavior, and classroom responsiveness	(x−1)/4
Content coverage	Alignment between lesson objectives, taught concepts, activities, and assessment content	(x−1)/4
Interaction quality	Relevance, timing, distribution, and pedagogical usefulness of teacher–student interaction	(x−1)/4
Feedback and adjustment	Diagnostic clarity, response to weak concepts, and adaptation of activity timing	(x−1)/4
Overall teaching quality	Holistic expert judgment after reviewing the lesson evidence	(x−1)/4

**Table 3:** Sensor-modality contribution calculated from 48 lesson-level observations. Pearson’s *r* is the correlation with the mean score of five expert raters; errors are lesson-level MAEs. The single-modality and fusion rows are implementation-level analog baselines aligned with recent classroom-analytics literature. The best result for each metric is shown in bold.

Method	Main Evidence	Pearson *r*	Engagement Error	Content Error
Rule-based	Head-up, interaction-count, and keyword-overlap rules	0.71	12.3%	14.5%
Visual only	Behavioral states	0.76	9.6%	13.8%
Acoustic-semantic only	ASR transcript and objectives	0.79	10.4%	8.1%
Interaction only	Learning-platform interaction logs	0.68	13.1%	12.7%
Visual + acoustic	Behavior and transcript semantics	0.84	7.4%	7.0%
SENSE	Visual, acoustic-semantic, interaction-log, and outcome-anchor evidence	**0.89**	**5.8%**	**6.2%**

**Table 4:** Robustness under missing-sensor simulation. Higher correlation and lower score drift indicate stronger stability.

Missing Setting	Rule *r*	SENSE *r*	Rule Drift	SENSE Drift
10% random	0.66	0.87	8.4%	3.2%
20% random	0.59	0.84	13.7%	5.1%
30% random	0.51	0.80	19.4%	7.8%
Visual masked	0.42	0.78	22.6%	8.9%
Acoustic masked	0.63	0.76	11.2%	10.4%

## Data Availability

Raw classroom videos, audio recordings, and individual learning-platform interaction logs are not publicly available because of participant privacy protection and institutional data-governance requirements. De-identified lesson-level features, prompt templates, aggregated statistics, and analysis code may be made available from the corresponding author upon reasonable request and subject to institutional approval.

## Supplementary Materials

The following supporting information can be downloaded at https://www.mdpi.com/article/10.3390/s26206617/s1, Supplementary Material S1: Reproducible Semantic-Evaluation Protocol for SENSE, including the prompt template, evidence-block definitions, JSON output schema, scoring protocol, expert-rating rubric, synthetic example, and prompt-version information.
