# Supplementary material for: SENSE: A Sensor-Centric Multimodal Evidence Chain for Teaching Evaluation in Smart Classrooms
Source: Sensors (Basel). 2026 Oct 19;26(20):6617. doi: 10.3390/s26206617 (PMC13708894; doi:10.3390/s26206617)
Supplement: Supplementary file 1 [file sensors-26-06617-s001.zip › sensors-4478637-supplementary.pdf]

Supplementary Material S1

Reproducible Semantic-Evaluation Protocol for SENSE

Lihong Cheng, Xiaohe Liu and Haisheng Yu

Supplement to "SENSE: A Sensor-Centric Multimodal Evidence Chain for Teaching Evaluation in Smart Classrooms"

S1. Purpose and Scope

This supplement documents the fixed semantic-evaluation protocol reported in the manuscript. It provides the prompt template, evidence-block definitions, output schema, scoring protocol, expert-rating rubric and examples, and prompt-version information needed to interpret and reproduce the SENSE evaluation procedure. No raw classroom video, raw audio, facial image, name, student identifier, teacher identifier, or individual-level record is included.

The prompt and examples below use placeholders or synthetic evidence. In the reported experiment, only de-identified lesson-level summaries and aggregated features were supplied to the semantic evaluator.

S2. Model and Prompt-Version Information

**Item**	**Fixed setting**
Prompt identifier	`SENSE-Prompt-v1.0`
Model identifier	`deepseek-chat`
Operating mode	Non-thinking mode
Temperature	0.1
`top_p`	1.0
Maximum output length	2048 tokens
Output constraint	JSON-constrained output
Streaming	Disabled
Repeated evaluations	Three runs per lesson using the same evidence packet
Aggregation	Median for continuous scores; majority agreement for categorical judgements
Score scale	Continuous dimension scores on a normalized 0–1 scale

S3. Evidence-Packet Definition

For lesson window t, the evaluator receives the aligned tuple

$$X_t = \{V_t, A_t, I_t, O_t\},$$

where V_t is visual behavioral evidence, A_t is acoustic-semantic transcript evidence, I_t is learning-platform interaction-log evidence, and O_t is available outcome-anchor evidence. Expert ratings are excluded from model inputs and are retained only as external validation references.

Each block contains an availability flag. Missing evidence must be reported as "not observed" and must not be converted into "observed as absent."

Evidence block	Included summaries	Interpretive restriction
Visual evidence	Active, passive, disengaged, and disruptive-state proportions; temporal changes; observable-frame coverage	Behavioral evidence is not treated as a direct measure of cognition.
Acoustic-semantic evidence	Key concepts, objectives, discourse focus, questions, responses, activity intervals, and relations between taught and assessed content	Transcript semantics are interpreted with the other evidence blocks, not as a standalone teaching score.
Interaction-log evidence	Timestamped question release, response submission, answer selection, task submission, quiz completion, feedback release, response density, and lesson-stage distribution	High event counts are not automatically interpreted as high-quality interaction.
Outcome anchors	Available lesson- or sequence-level assessment and response summaries	Outcomes provide contextual anchors and are not sensor modalities or proof of causality.

S4. Fixed Prompt Template

The following template documents the semantic-evaluation instruction and the four evidence blocks used for each lesson. Bracketed fields are replaced with de-identified, aggregated lesson evidence.

```
SYSTEM ROLE
You are an educational-evaluation assistant. Evaluate the lesson only
from the structured evidence supplied below. Do not infer identities,
emotions, cognitive states, or events that are not supported by the
evidence. Treat missing evidence as unavailable, not as negative evidence.

EVALUATION RULES
1. Use all available evidence blocks and explicitly identify unavailable
   blocks.
2. Distinguish observable classroom behavior from cognitive engagement.
3. Do not treat interaction frequency alone as interaction quality.
4. Evaluate content coverage conceptually, not only through keyword overlap.
5. Every evaluative judgement must cite at least one evidence reference.
6. State uncertainty when evidence is incomplete, sparse, or conflicting.
7. Return valid JSON matching the required schema. Do not add text outside
   the JSON object.

LESSON METADATA
lesson_id: [DE-IDENTIFIED LESSON IDENTIFIER]
analysis_window: [START-END OR LESSON-LEVEL SUMMARY]
prompt_version: SENSE-Prompt-v1.0

BLOCK V: VISUAL EVIDENCE
availability: [available/unavailable/partial]
observable_frame_coverage: [0-1]
active_engagement_proportion: [0-1 or null]
passive_engagement_proportion: [0-1 or null]
disengagement_proportion: [0-1 or null]
disruptive_behavior_proportion: [0-1 or null]
temporal_summary: [DE-IDENTIFIED AGGREGATED SUMMARY]

BLOCK A: ACOUSTIC-SEMANTIC EVIDENCE
availability: [available/unavailable/partial]
lesson_objectives: [LIST]
key_concepts: [LIST]
questions_and_responses: [AGGREGATED SUMMARY]
```

```
activity_intervals: [AGGREGATED SUMMARY]
assessment_content_relation: [STRUCTURED SEMANTIC SUMMARY]

BLOCK I: LEARNING-PLATFORM INTERACTION-LOG EVIDENCE
availability: [available/unavailable/partial]
event_counts: [AGGREGATED COUNTS]
response_density: [VALUE OR null]
timing_summary: [SUMMARY]
submission_summary: [SUMMARY]
lesson_stage_distribution: [SUMMARY]

BLOCK O: OUTCOME ANCHORS
availability: [available/unavailable/partial]
assessment_summary: [DE-IDENTIFIED AGGREGATED SUMMARY]
sequence_or_order_context: [SUMMARY]
limitations: [SUMMARY]

TASK
Produce scores for engagement, content coverage, interaction quality,
and overall teaching quality. Provide concise evidence-grounded feedback,
evidence references, and a confidence explanation. Follow the scoring
rules and JSON schema in this supplement.
```

S5. JSON Output Schema

Scores are continuous values from 0 to 1. Evidence references use the block identifiers `V`, `A`, `I`, and `O`. A reference may additionally identify a lesson window or a summary field.

```
{
  "lesson_id": "string",
  "prompt_version": "SENSE-Prompt-v1.0",
  "scores": {
    "engagement": 0.0,
    "content_coverage": 0.0,
    "interaction_quality": 0.0,
    "overall_teaching_quality": 0.0
  },
  "judgements": {
    "engagement": "string",
    "content_coverage": "string",
    "interaction_quality": "string",
    "overall_teaching_quality": "string"
  },
  "evidence_references": {
    "engagement": ["V:field/window", "I:field/window"],
    "content_coverage": ["A:field/window", "O:field"],
    "interaction_quality": ["A:field/window", "I:field/window"],
    "overall_teaching_quality": ["V/A/I/O references"]
  },
  "feedback": [
    {
      "issue": "string",
      "recommended_action": "string",
      "evidence_references": ["block:field/window"]
    }
  ],
  "confidence": {
    "level": "high|moderate|low",
    "explanation": "string",
    "unavailable_or_partial_blocks": ["V", "A", "I", "O"]
  }
}
```

The output is rejected and regenerated when it is not valid JSON, omits a required field, produces a score outside 0–1, or gives an evaluative judgement without an evidence reference.

S6. Semantic-Evaluation Scoring Protocol

Dimension	Evidence considered	Scoring interpretation
Engagement	Observable behavior, valid-frame coverage, speaking or response events, and relevant platform interactions	Higher scores require converging evidence of sustained participation. Head-up behavior alone is insufficient. Missing or occluded observations reduce confidence rather than automatically reducing engagement.
Content coverage	Lesson objectives, transcript concepts, instructional activities, assessment or activity content, and available outcome anchors	Higher scores indicate conceptual alignment among objectives, taught content, activities, and assessment evidence. Surface keyword overlap alone is insufficient.
Interaction quality	Relevance, timing, distribution, response density, questions and responses, and pedagogical usefulness	Higher scores require relevant and appropriately timed exchanges distributed across the lesson. Event frequency alone cannot determine quality.
Overall teaching quality	Joint interpretation of engagement, coverage, interaction, feedback opportunities, evidence completeness, and contextual outcome anchors	This is a holistic judgement constrained by the component evidence and its limitations; it is not a simple unweighted average.
Feedback	Low-engagement windows, weakly covered concepts, low-response intervals, timing problems, and confidence limitations	Feedback must be specific, actionable, and linked to cited evidence. It must not introduce unsupported events or causal claims.
Confidence explanation	Availability, completeness, temporal alignment, consistency, and conflict among evidence blocks	Confidence is high for complete and convergent evidence, moderate for partial but interpretable evidence, and low for substantial missingness or unresolved conflict.

S7. Expert-Rating Rubric

Five university English teachers independently evaluated the same 48 lessons without access to SENSE or baseline outputs. Each item was scored on a five-level ordinal scale and normalized as $(x - 1)/4$, where $x \in \{1, 2, 3, 4, 5\}$. The lesson-level expert reference was the mean of the five normalized expert ratings.

Dimension	Evaluation focus
Engagement evidence	Visible attention, active participation, off-task behavior, classroom responsiveness, and observation coverage.
Content coverage	Alignment among lesson objectives, taught concepts, instructional activities, and assessment content.
Interaction quality	Relevance, timing, distribution, and pedagogical usefulness of teacher–student interaction.
Feedback and adjustment	Diagnostic clarity, response to weak concepts, and adaptation of questioning or activity timing.
Overall teaching quality	Holistic expert judgement after reviewing the available lesson evidence.

Shared Five-Level Anchors

Level 1: Evidence indicates very weak performance on the dimension, with major deficiencies across the lesson.

Level 2: Evidence indicates weak performance, with substantial limitations and only isolated strengths.

Level 3: Evidence indicates acceptable or mixed performance, with both strengths and limitations.

Level 4: Evidence indicates strong performance, with minor limitations that do not dominate the lesson.

Level 5: Evidence indicates consistently strong performance across relevant lesson stages, supported by clear evidence.

Experts were instructed to mark a dimension as not rateable when the evidence needed for that dimension was unavailable. A missing rating was not converted to the lowest score.

S8. Synthetic Scoring Example

The following example is synthetic and is included only to demonstrate application of the protocol.

Synthetic evidence summary. Visual evidence is available for 88% of sampled frames and indicates mostly passive engagement with two low-response windows. The transcript covers four of five target concepts and includes questions on three concepts. Platform logs show responses concentrated in the middle of the lesson and few responses during the final activity. The outcome anchor is available only at lesson-sequence level.


```
{
  "lesson_id": "SYNTHETIC-EXAMPLE",
  "prompt_version": "SENSE-Prompt-v1.0",
  "scores": {
    "engagement": 0.66,
    "content_coverage": 0.80,
    "interaction_quality": 0.62,
    "overall_teaching_quality": 0.71
  },
  "judgements": {
    "engagement": "Participation was generally sustained but was mainly passive, with two low-
    response windows.",
    "content_coverage": "Most target concepts were taught and linked to activities; one target
    concept was weakly represented.",
    "interaction_quality": "Questions were relevant, but responses were unevenly distributed across
     lesson stages.",
    "overall_teaching_quality": "The lesson showed good content alignment with moderate engagement
    and interaction limitations."
```

```
  },
  "evidence_references": {
    "engagement": ["V:coverage_and_states", "I:response_timing"],
    "content_coverage": ["A:key_concepts", "O:sequence_summary"],
    "interaction_quality": ["A:questions_and_responses", "I:lesson_stage_distribution"],
    "overall_teaching_quality": ["V:summary", "A:summary", "I:summary", "O:summary"]
  },
  "feedback": [
    {
      "issue": "Low response during the final activity",
      "recommended_action": "Add a targeted check-for-understanding before the final task closes.",
      "evidence_references": ["I:lesson_stage_distribution", "V:low_response_windows"]
    }
  ],
  "confidence": {
    "level": "moderate",
    "explanation": "The three process-evidence blocks are available, but the outcome anchor is
    sequence-level rather than window-level.",
    "unavailable_or_partial_blocks": ["O"]
  }
}
```


S9. Fixed Evidence-Processing Parameters

The following implementation settings were fixed before final lesson-level scoring:

- video sampling rate: 2 frames per second;
- non-overlapping analysis-window length: 30 seconds;
- head-up yaw threshold: absolute yaw not exceeding 35°;
- head-up pitch range: $-20°$ to $25°$;
- disengagement threshold: off-task, head-down, or off-screen behavior occupying more than 60% of valid frames in a window;
- valid student-window threshold: at least 50% of expected sampled frames observable and alignable with the lesson timeline;
- missing-sensor stress tests: random masking of 10%, 20%, and 30% of evidence units, plus complete masking of visual or acoustic evidence;
- predictive ablation sequence: V, $V + A$, $V + A + I$, and $V + A + I + O$.

S10. Privacy and Use Restrictions

This supplement documents the evaluation protocol but does not release restricted classroom data. Raw recordings and individual learning-platform interaction logs remain unavailable because of participant privacy and institutional data-governance requirements. The template must be populated only with de-identified, aggregated evidence. Semantic-evaluator outputs require human review and must not be treated as autonomous high-stakes decisions about individual teachers or students.
